# Identification of GINS2 prognostic potential and involvement in immune cell infiltration in hepatocellular carcinoma

**DOI:** 10.7150/jca.53841

**Published:** 2022-01-01

**Authors:** Zuyin Li, Guohe Song, Dezhen Guo, Zhijie Zhou, Chen Qiu, Chao Xiao, Xiaoliang Wang, Yupeng Wang

**Affiliations:** 1 Department of Hepatobiliary Surgery, Peking University Organ Transplantation Institute, Peking University People's Hospital, Beijing, 100034, China.; 2 Department of General Surgery, Shanghai General Hospital, Shanghai Jiao Tong University School of Medicine, Shanghai, 201600, China.; 3 Department of Liver Surgery and Transplantation, Liver Cancer Institute, Zhongshan Hospital, Fudan University; Key Laboratory of Carcinogenesis and Cancer Invasion (Ministry of Education), Fudan University, Shanghai, 200032, China.; 4 Department of General Surgery, Huashan Hospital North, Fudan University, Shanghai 201907, China.; 5 Institute of Gallstone Disease, Center of Gallbladder Disease, Shanghai East Hospital, Tongji University School of Medicine, Shanghai, 200120, China.; 6 Department of General Surgery, Huashan Hospital, Fudan University, Shanghai, 20040, China.; 7 Department of General Surgery, Qingpu Branch of Zhongshan Hospital Affiliated to Fudan University, Shanghai, 201700, China.

**Keywords:** GINS2, DNA replication, cell cycle, immune cell infiltration.

## Abstract

**Background**: *GINS2* has been reported to have prognostic value in several solid tumors other than hepatocellular carcinoma (HCC), and its influence on tumor immunity has not been investigated thus far.

**Methods**: The transcriptome profiles were retrieved from two public databases, GEO and TCGA. The median *GINS2* expression was considered as cutoff to define *GINS2*^high^ and *GINS2*^low^ groups and to obtain differentially expressed genes. These genes were then subjected to KEGG pathway and gene ontology (GO) analysis and to gene set enrichment analysis (GSEA). Survival analyses according to *GINS2* level were performed utilizing Kaplan-Meier plotter. TIMER database was adopted to investigate associations between *GINS2* level and infiltrating immunocytes, and the correlation between immunocyte-related gene expression and *GINS2* level was evaluated via GEPIA database. A 236-patient validation cohort were applied to confirm the bioinformatic results of TCGA and TIMER database.

**Results**: *GINS2* is augmented in tumorous tissues of HCC patients compared with nontumor specimens, and GINS2-overexpressed patients have poorer overall survival (OS) and disease-specific survival (DSS) than those with low GINS2 expression in HCC (*P* = 0.009 and *P* = 0.002 respectively). Cell cycle and DNA replication were two main processes that enriched in tumor cells overexpressed *GINS2* gene (NES = 1.848, *P* = 0.007; and NES = 1.907, *P* = 0.005, respectively). Moreover, *GINS2* correlates positively with markers of activated CD8^+^ and CD4^+^ T cells, as well as exhausted T lymphocytes.

**Conclusions**: HCC patients overexpressed *GINS2* have poorer prognoses than those with low *GINS2* expression, possibly as a result of the function of *GINS2* in cell cycle and DNA replication as well the exhaustion of T lymphocytes.

## Introduction

Hepatocellular carcinoma is currently the sixth of top ten incidence of malignant tumor among both males and females worldwide but the fourth deadly disease of cancer 1. Accumulating evidence reveal that the biological behavior of cancer cells is tightly related with diverse elements of tumor microenvironment (TME), say, immune cells. They function as key factors in TME and are of great significance in tumorigenesis and progression 2. With the gradual understanding of TME in HCC, the appropriate stratification in patients may gain great benefit from targeted immunotherapies 3.

When tumor cells are growing and prosper, they seek to compromise immune surveillance within the microenvironment 4. The molecules, say, PD-1/PD-L1 interaction, act as a key role in this process 5. Researchers noticed that the levels of PD-1 in CD8^+^ T cells was elevated in patients with liver cancer, and analyses of samples from patients who have not obtained any medical treatment show the infiltrating abundance of PD-1^+^ CD8^+^ T cells is related with poor outcomes 6-8. Moreover, it has also been discovered in HCC patients that the overexpressed PD-L1 on cancerous cells can lead to T cell exhaustion and functional incompetence 9. Hence, the goal of immunotherapy is to activate the patient's own incompetent or suppressed immune system to generate antitumor effects. Increasing evidence has demonstrated that dysregulated gene expression is correlated with immune abnormalities, as well as dysfunction of multiple biological processes, including dysregulated cell cycle and DNA replication processes, although the complex regulatory network involving these genes is still unknown. Therefore, in-depth analysis of these genes will help understand the mechanistic basis for tumorigenesis and development of HCC and clarify the interaction between the molecules and immune infiltration.

GINS complex subunit 2 (*GINS2*), systematically identified in 2003, is an essential protein for GINS, a pivotal complex for the processes of DNA replication 10. *GINS2* can bind preferentially to single-stranded DNA, which is especially significant to ensure the orderly progress of DNA replication in many cell types and organs 11. In 2009, *GINS2* was first suggested as a crucial gene promoting metastasis in breast cancer 12. Previous studies indicate that downregulation of *GINS2* causes decreased cell vitality, promotes apoptosis and the inhibition cell cycle progression in pancreatic tumor cells 13. These evidence hints that *GINS2* functions as a pivotal molecule in tumorigenesis. In addition, some reports have revealed that *GINS2* overexpression bring unfavorable outcomes in different tumor types, such as non-small-cell lung cancer (NSCLC), breast and cervical cancer 14-16. However, there are few reports on the utility of *GINS2* as a biomarker for the prognosis of HCC and its involvement in immune infiltration. Moreover, the underlying functional and mechanistic basis of *GINS2* in tumor proliferation and TME remain unknown.

In the present study, we identified *GINS2* overexpression in HCC patients and its impact on the clinical outcomes of HCC patients and explored the association of *GINS2* with immunocyte infiltration as well as immune marker gene sets via TIMER and GEPIA database as well as a 236-patient validation cohort. Our results demonstrated the significance of *GINS2* in the occurrence and progression of hepatocellular carcinoma and illustrated the association between the levels of *GINS2* and prognosis as well as immune cell infiltration.

## Materials and methods

### Data resources and descriptions

Five RNA-sequencing datasets (The sample size exceeds 100 cases), GSE77314, GSE45436, GSE36376, GSE25097 and GSE14520, including HCC tumorous and nontumor specimens were retrieved from Gene Expression Omnibus (GEO, https://www.ncbi.nlm.nih.gov/geo/). The details of each GEO datasets are presented in Table S1. The raw count data and clinical information of TCGA-LIHC cohort were obtained from Genomic Data Commons Data Portal (https://cancergenome.nih.gov/). The RNA-seq dataset contains 421 samples, including 371 tumorous tissues and 50 normal liver specimens. The “edgeR” package is applied to analyze the levels of *GINS2* between tumorous and nontumor specimens.

### Patients and tissue samples

To prove the results from database, we collected 236 patients with HCC to form a validation cohort. This cohort consisted of 236 patients who were diagnosed with HCC in the Department of Liver Surgery and Transplantation, Zhongshan Hospital, Fudan University, between 2007 and 2008. Clinical information was summarized from electronic medical records. Tissue samples collected during surgery were pathologically examined, fixed in 4% paraformaldehyde and stored at the tissue bank before analysis. The study was approved by the Ethical Committees of Zhongshan Hospital, and each subject was fully informed and signed the written consent.

### Immunohistochemical (IHC) staining and evaluation of GINS2 protein expression

Sections of HCC samples and adjacent normal specimens were incubated with a rabbit polyclonal anti-GINS2 antibody (1:200, #ab197123, Abcam, Cambridge, UK). The results of IHC staining were examined under double-blinded conditions and scored on a semi-quantitative scale based on the score of intensity and extent. The score of intensity was marked as 0 (no staining), 1 (weak), 2 (moderate), and 3 (strong), and the percentage of positive stained area as 0 (none), 1 (1-25%), 2 (26-50%), 3 (51-75%), and 4 (>=75%). The IHC score was calculated by multiplying the score of intensity and extent in each sample (scale range 0-12). The levels of GINS2 were divided into “GINS2 Low” group (score <6) and “GINS2 High” group (scores >=6).

### Multiplex immunohistochemistry (mIHC)

TSA plus fluorescent multiple staining kit (#G1236-100T, Servicebio) was applied for multiplex IHC. The 5-mm formalin-fixed and parrffin-embedded slides were deparaffinized and rehydrated. All slides were subjected to epitope retrieval with EDTA (pH 8.0) for 8 min. After cooling (25℃), slides were washed with PBS (3*5 min), and endogenous peroxidase activity were blocked with H_2_O_2_ (3%) for 25 min. Then blocking buffer (5%BSA, Solarbio, #SW3015) was used for 30 min-protein blocking. All slides were incubated with antibody against CD8 (#GB13068, Servicebio, 1:500) at 4℃ overnight. After washes, sections were incubated with an HRP-conjugated secondary antibody for 50 min. Then slides were dyed with CY3-TSA for 10 min. This method was applied three more times using the antibodies as follows, GINS2 (Proteintech, #16247-1-AP, 1:1000, dye FITC), CD4 (Wisee Biotechnology, #YX32005-100, 1:1000, dye 647-TSA), and CD3 (Servicebio, #GB13440, 1:100, dye594). EDTA (pH 8.0) buffer was applied for the next round of epitope retrieval with a cooker (125℃, 15 min). Cell nucleus was labeled with DAPI (Servicebio, #G1012) and covered with Antifade Mounting Medium (Beyotime, #P0126). Secondary antibodies were used as follows: anti-rabbit (1:500, Servicebio, GB23303) and anti-mouse (1:500, Servicebio, GB23301).

### Differentially expressed gene (DEGs) analysis

In the TCGA dataset, we analyzed DEGs by applying the “edgeR” package between GINS2^high^ and *GINS2*^low^ group defined by the median expression of *GINS2*. Fold change >2 and adjusted *P* value (Benjamini and Hochberg adjustment) < 0.05 were used as the cutoffs for DEGs. To determine the functions of the 421 upregulated DEGs, these genes were then subjected to DAVID 6.8 (https://david.ncifcrf.gov/) for KEGG pathway and GO functional analyses and via Cytoscape software for result visualization, as summarized by ClueGo modules. Correlations between *GINS2* and cell cycle- or DNA replication-related genes were determined by Prism 8 software with Pearson's R and *P* values.

### Kaplan-Meier plotter database analysis

Kaplan-Meier plotter can evaluate the impact of 54,000 genes (encoding mRNA, miRNA, and protein) on the prognosis of 21 tumor types, including HCC (n=364). The relationship between the levels of *GINS2* and survival in HCC was evaluated via the liver cancer module (http://kmplot.com/analysis/)^17^. Hazard ratio (HR), 95% confidence interval (CI), and log-rank P-value were presented. For subgroup analysis, we chose all default settings and applied the survival analysis.

### Immune infiltration analysis

TIMER database provides resources to comprehensively investigate the immune infiltration of all tumor types (https://cistrome.shinyapps.io/timer/) 18. Analysis of *GINS2* expression level was performed in multiple cancer types via the DiffExp module, and the association between the *GINS2* levels and infiltrating immunocytes was determined through gene modules. The correlation modules can assess the relation between *GINS2* levels and markers of tumor-infiltrating immune cells. We chose the immune-related markers presented in previous studies 19-21. The correlation module was utilized to draw expression scatterplots, and Spearman's rho value was calculated to estimate statistical significance.

### Correlation analysis from GEPIA database

GEPIA database is a multifunctional web tool that contains 9,736 tumor and 8,587 normal specimens from the TCGA and GTEx database, which analyze RNA-seq profiles 22. In this study, the tool was applied to further verify the association between GINS2 and marker gene sets of monocytes and Tumor-associated macrophages (TAMs). The Pearson method was adopted to calculate the correlation coefficient. The analysis include tumor and nontumor tissue datasets. The R and *P* values for the correlation between GINS2 and genes of interest generated by GEPIA are summarized in Table 4.* P* <0.05 was considered statistically significant.

### Gene set enrichment analysis (GSEA)

To discover potential biological differences among high- and low-GINS2 expression in the liver of HCC patients, we utilized GSEA to obtain relevant pathways using GSEA. As mentioned before, we split all HCC patients into GINS2^high^ and GINS2^low^ groups. The ranks of all genes were obtained through their differences between two groups. GSEA concentrates on the expression data at the levels of specific gene sets. The normalized enrichment score (NES) was calculated. We chose 1000 times in the process of the gene set permutations and set a* P*-value cutoff of 0.05.

### Statistical analysis

Differences in the transcriptional levels of gene among the defined groups were evaluated via Student's t-test or Mann-Whitney U-test based on variable type. Chi-squared test was utilized to evaluate the relation between GINS2 levels and clinicopathological parameters. Statistical software (SPSS, version 25.0) from SPSS Inc. (Chicago, IL, USA) was used. A two-tailed *P* <0.05 was considered significant for all analyses.

## Results

### Upregulated *GINS2* in tumor tissues predicts poorer prognoses of HCC patients

**Figure 1:**
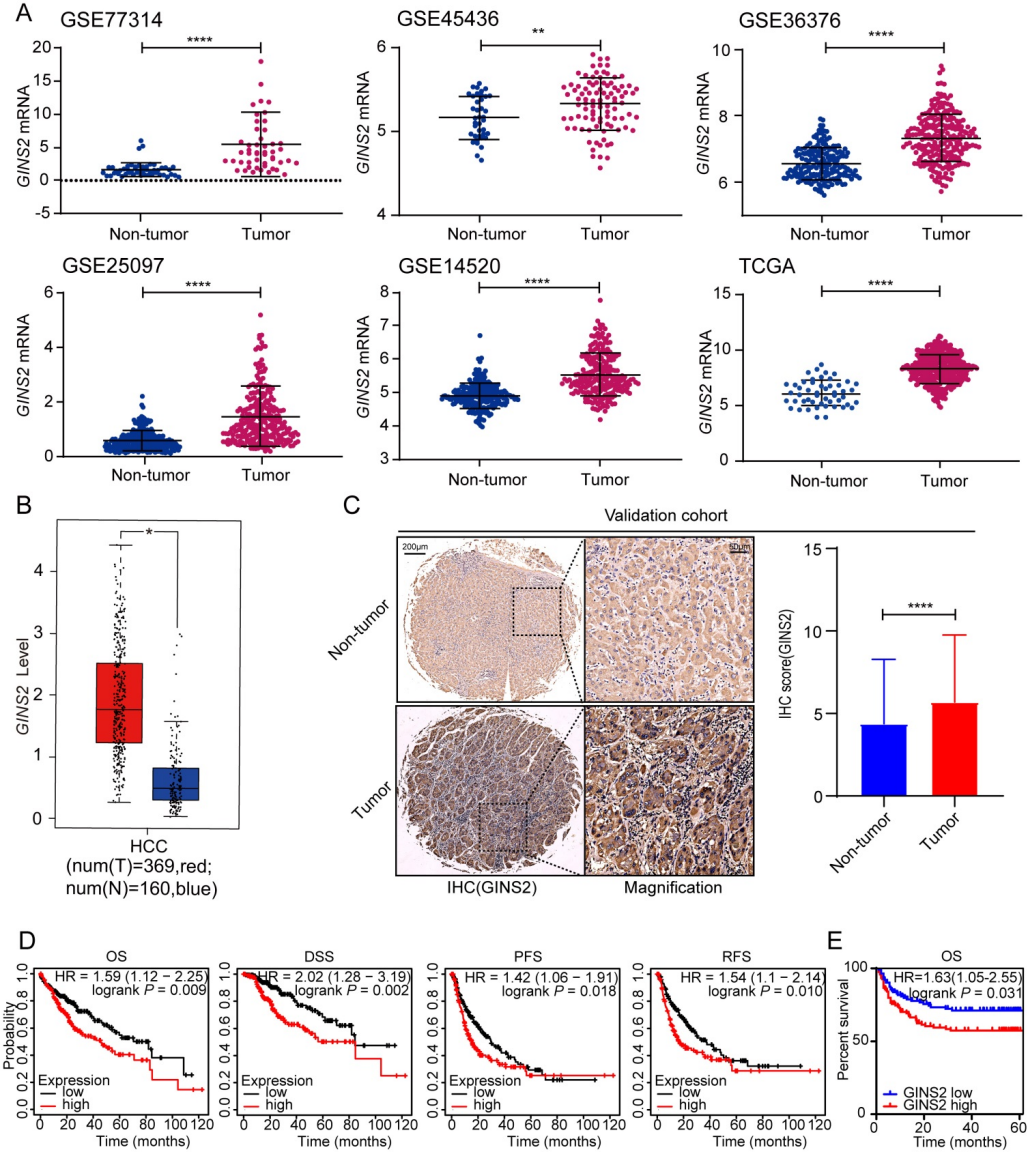
** Up-regulated *GINS2* in tumorous tissues and predicts poorer prognoses of HCC patients.** A. *GINS2* mRNA expression levels between tumor and non-tumor tissues of HCC patients of GEO series and TCGA database by Student's *t*-test or Mann-Whitney *U* test. B. Box plot of *GINS2* levels in tumor and adjacent tissues from the GEPIA database. C. Representative immunohistochemistry staining and statistical results reveal the protein levels of *GINS2* in the validation cohort. D. Survival analyses of HCC cohort from TCGA database grouped by *GINS2* expression utilizing the K-M plotter tool. OS, overall survival; DSS, disease specific survival; PFS, progression-free survival; RFS, Relapse-free survival. E. Kaplan-Meier survival analysis of the relationship between *GINS2* protein expression and OS in the validation cohort. *, *P* < 0.05, **, *P* < 0.01, ***, *P* < 0.001, ****, *P* < 0.0001.

To evaluate the transcriptional levels of *GINS2* gene in tumor and nontumor tissues, five GEO expression microarray datasets and expression profiles from the TCGA-LIHC cohort were used. By contrast, *GINS2* gene was significantly overexpressed in tumor samples of the GSE77314, GSE45436, GSE36376, GSE25097, GSE14520 and TCGA datasets (all P < 0.01, Figure 1A). Moreover, the transcriptional data from the GEPIA database also confirmed the overexpression of *GINS2* in tumor samples (T, red, Figure 1B). To further prove these results in the database, we performed IHC staining of tissues from a 236-patient validation cohort. The IHC analysis showed that the protein expression of *GINS2* in tumor samples was significantly higher than those in matched nontumor tissues (Figure 1C). Next, we examined the *GINS2* expression in TIMER database, which extracts expression profiles of multiple malignancies from TCGA. *GINS2* expression was undoubtedly higher in HCC tumor samples than in normal specimens and showed a pan-cancer overexpression signature (Figure S1). Collectively, these results showed significant overexpression of *GINS2* in HCC patients.

To explore the impacts of *GINS2* overexpression on HCC prognosis, we determined the correlation between clinicopathological characteristics and *GINS2* expression status in the TCGA cohort. We split the TCGA-LIHC cohort equally in two groups. Patients with *GINS2* expression higher than the median expression value of *GINS2* were deemed as “high” group and the rest, "low" group. The results revealed a tight correlation between *GINS2* overexpression and T stage as well as pathologic stage of HCC patients (Table 1, all *P* < 0.05). Our validation cohort also confirmed that the overexpression of GINS2 was related with TMN stage (Table S2). Furthermore, we analyzed the levels of *GINS2* expression in TCGA cohort to evaluate the role of *GINS2* gene in the outcomes of patients. Overall survival (OS, *P* = 0.009), disease-specific survival (DSS, *P* = 0.002), progression-free survival (PFS, *P* = 0.018) and relapse-free survival (RFS, *P* = 0.010) analysis showed that *GINS2* overexpression was generally correlated with shorter survival time and poorer prognosis in HCC patients (Figure 1D). The result of OS (*P* = 0.009) was further verified in the validation cohort (Figure 1E). We additionally applied subgroup survival analysis in different populations. The results revealed that *GINS2* overexpression contributed to worse OS and DFS in male HCC cohort, and a high *GINS2* level caused unfavorable OS and DSS in patients without a history of alcohol consumption or hepatitis virus infection (Figure S2A and S2B). Moreover, high *GINS2* expression was a risk factor for OS and DSS in Asian HCC patients (Figure S2A and S2B). Collectively, the above evidences demonstrate that *GINS2* overexpression is significantly associated with unfavorable survival outcomes in HCC patients.

### *GINS2* is vital in the processes of cell cycle and DNA replication

**Figure 2:**
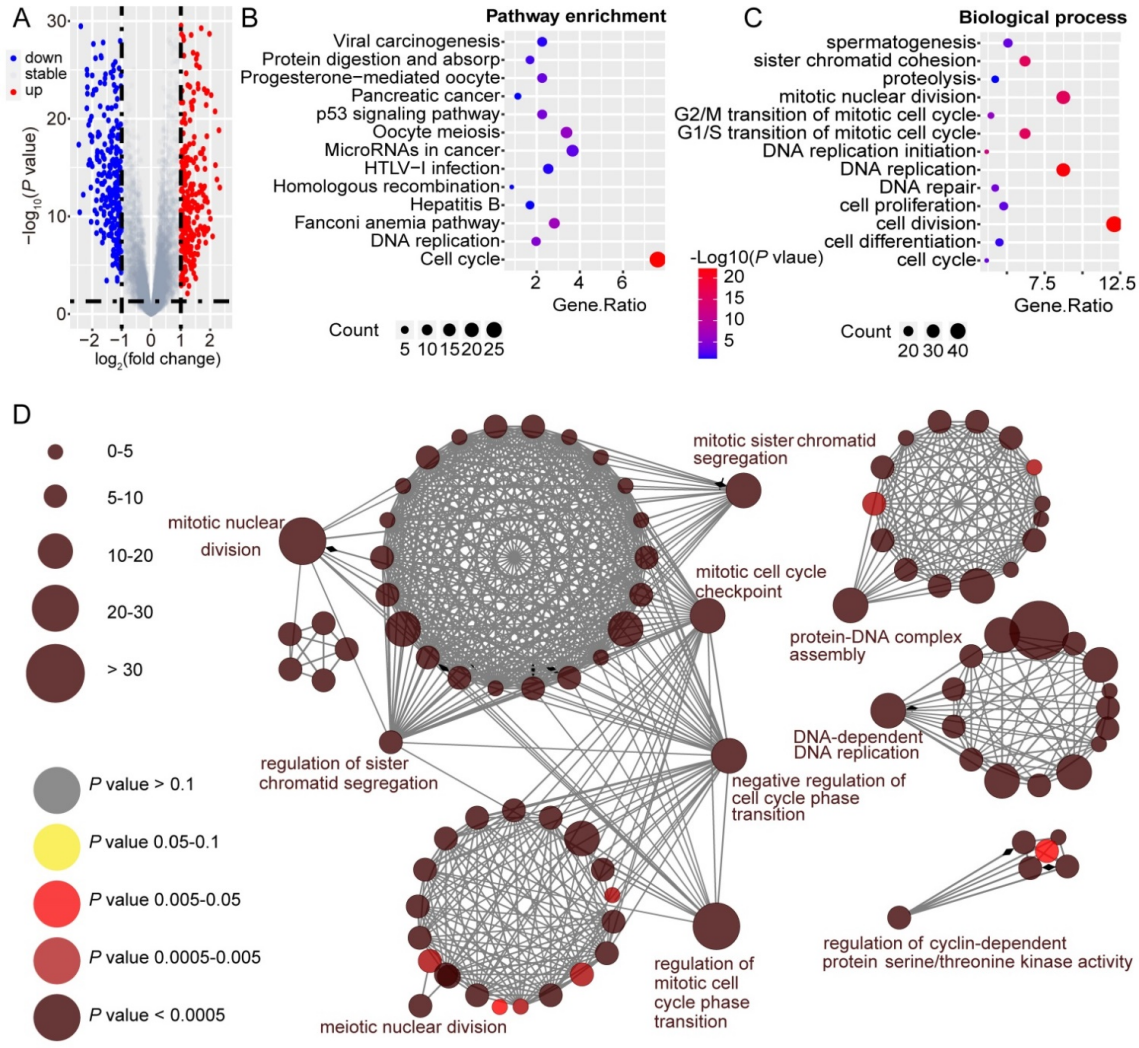
***GINS2* is vital in the processes of cell cycle and DNA replication.** A. Volcano plot indicates DEGs by comparing *GINS2*-high and *GINS2*-low samples from the TCGA database. Blue dot, down-regulated DEGs; red dot, up-regulated DEGs. B/C. Pathway enrichment and biological process of 412 up-regulated DEGs by KEGG analysis. D. Visualization of the interaction network of DEGs by Cytoscape (Cluego module). Node size indicates the mapped gene number; the node color schedule represents the *P* value.

To unravel the underlying role of *GINS2* in liver carcinogenesis and tumor progression, we compared DEGs in the TCGA dataset between GINS2^high^ and GINS2^low^ groups determined by the *GINS2* median cutoff. KEGG pathway and GO analysis were utilized to explore the function of these DEGs (the cutoff criteria were log2(FC)>1). Within these DEGs, 421 genes were upregulated, and 280 genes were downregulated (Figure 2A). KEGG pathway analysis demonstrated that the upregulated DEGs were mainly enriched in cell cycle and DNA replication signaling pathways (Figure 2B). GO analysis hinted that biological processes involving these upregulated DEGs were cell division, DNA replication and mitotic nuclear division, processes that are related to tumorigenesis and growth (Figure 2C). KEGG pathway analysis also demonstrated that the downregulated DEGs were mainly about metabolic pathways, and the enriched biological processes included oxidation-reduction processes (Figure S3A and S3B). The interactive association of these upregulated DEGs and corresponding term clusters were shown by ClueGO to decipher the biological processes and associations of functionally grouped genes (Figure 2D). The interaction network of DEGs included 139 representative terms and 446 term connections. The results revealed that the significantly enriched biological processes were cell cycle, DNA replication, and processes that have been deemed essential preparations for cell proliferation.

**Figure 3:**
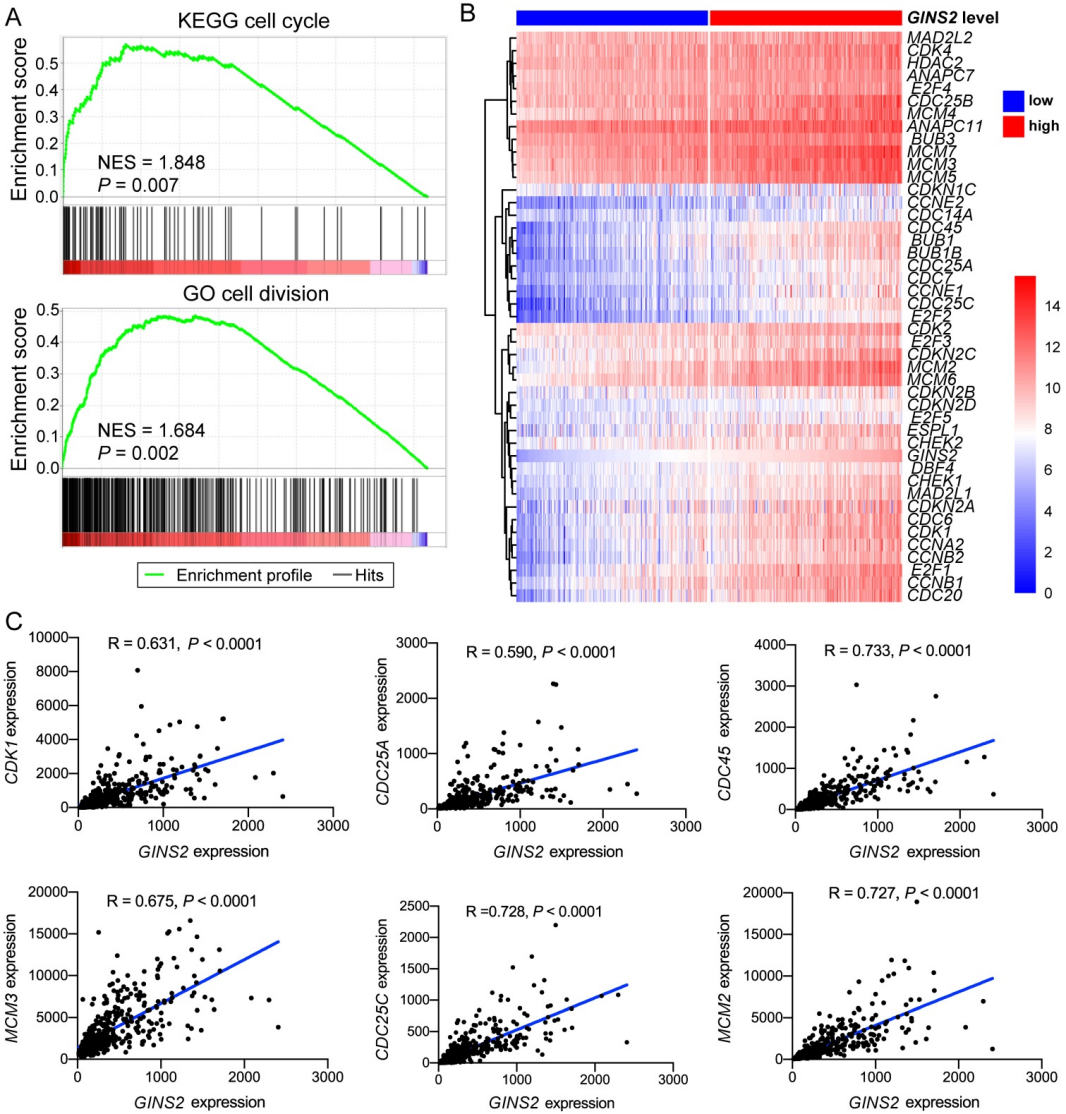
** Associations between *GINS2* and key genes related to cell cycle in HCC.** A. GSEA analysis prompts *GINS2* is positively related to cell cycle and cell division. B. Heatmap of genes related to cell cycle grouped by *GINS2*-high and *GINS2*-low samples. C. Pearson correlation analysis of *GINS2* and key genes involved in cell cycle.

GSEA analysis was also utilized to identify the pathways significantly correlated with *GINS2* levels (*GINS2*^high^ vs. *GINS2*^low^). The results demonstrated that the gene sets related with high *GINS2* expression were enriched in cell cycle, cell division, chromosome separation and DNA replication (Figure 3A, Figure S4A). To further explore the associations between *GINS2* and cell cycle processes in HCC, a panel of genes related to the cell cycle was compared between the two *GINS2* expression groups (*GINS2*^high^ vs. *GINS2*^low^), and the results indicated that most of these genes were upregulated in the *GINS2*^high^ group. Furthermore, *CDK1*, which acts as a key factor in the process of the eukaryotic cell cycle by regulating the centrosome cycle and mitotic onset; *CDC25A*, which takes effect by inducing mitotic progression; and other genes related to the cell cycle (e.g., *CDC45*, *MCM3* and *CDC25C*) and DNA replication (e.g., *CDT1*, *CHAF1A*, and *FEN1*) were positively correlated with the expression of *GINS2* (Figure 3C, Figure S4B), which further implied the significant role of *GINS2* in liver carcinogenesis and tumor progression.

### *GINS2* and its co-expressed genes show significant associations with the infiltrating abundance of immune cells in HCC

**Figure 4:**
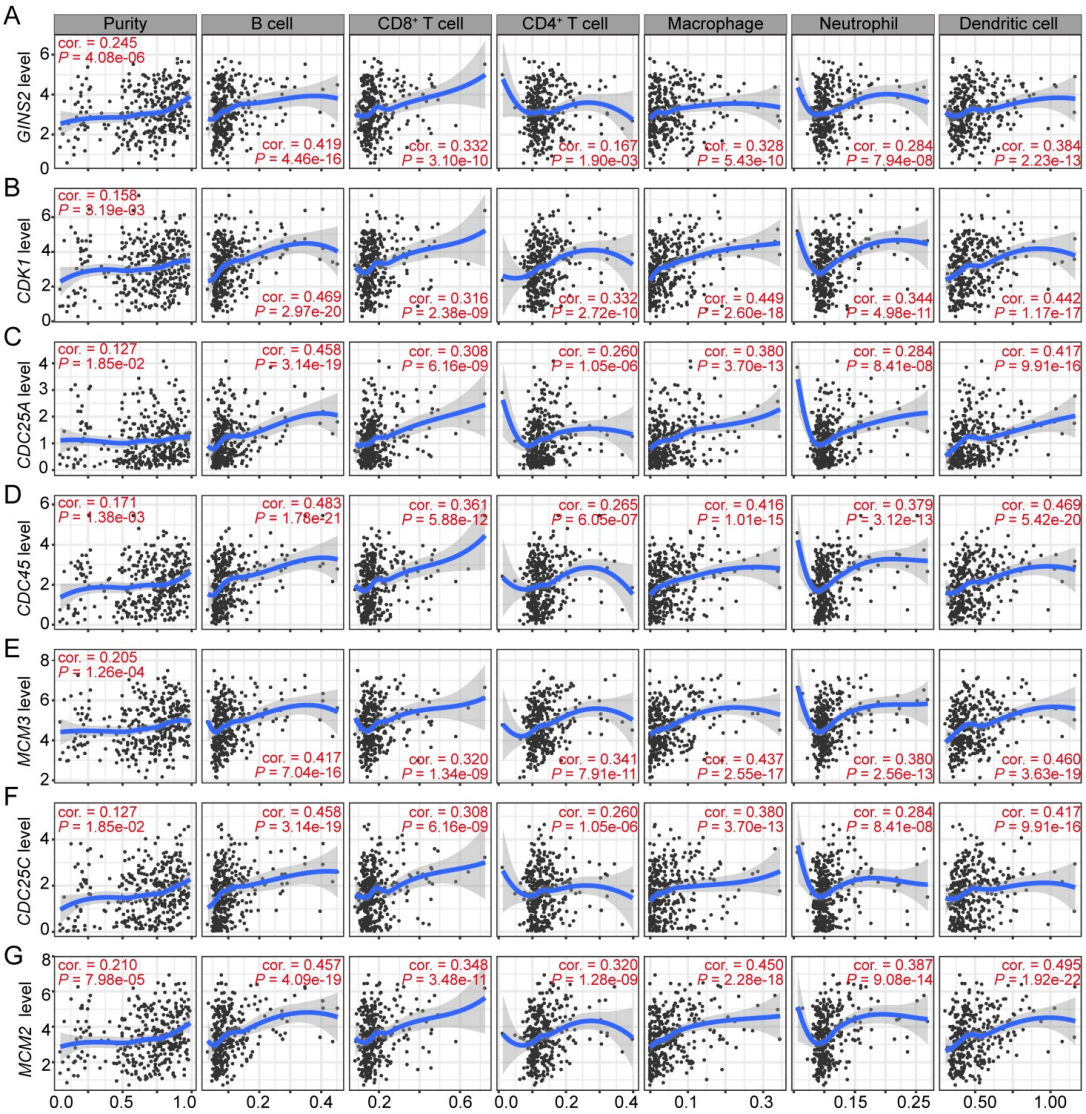
** Significant correlations between *GINS2* and its co-expressed cell cycle related genes and immunocytes infiltration levels in HCC.** Associations between *GINS2* (A), CDK1 (B), CDC25A (C), CDC45 (D), MCM3 (E), CDC25C (F), MCM2 (G) expression and diverse immune cells infiltration in HCC. Cor, R value of Spearman's correlation.

Immune cells comprise the most of tumor microenvironment (TME) and somehow participate in tumor cell proliferation and development 23-25. Therefore, we sought to explore whether the transcription level of *GINS2* was associated with the infiltrating abundance of immune cells in HCC. The results revealed that *GINS2* levels was positively associated with the abundance of different infiltrating immune cells, including B cells, CD8^+^ T cells, macrophages, neutrophils, and dendritic cells (DCs), in HCC (Figure 4A). Moreover, *GINS2* co-expressed and cell cycle-related genes, such as* CDK1*, *CDC25A*, *CDC45*, *MCM3*, *CDC25C* and *MCM2*, were also shown positive correlation with the abundance of infiltrating B cells, CD8^+^ T cells, CD4^+^ T cells, macrophages, neutrophils and DCs in HCC (Figure 4B-G). Altogether, these above evidences suggest that GINS2 and its co-expressed genes may have an influence in the immune response in TME by affecting immune infiltrating abundance, especially those of B cells, CD8+ T cells, macrophages and DCs.

### GINS2 levels and its correlation with immune marker gene sets

**Figure 5:**
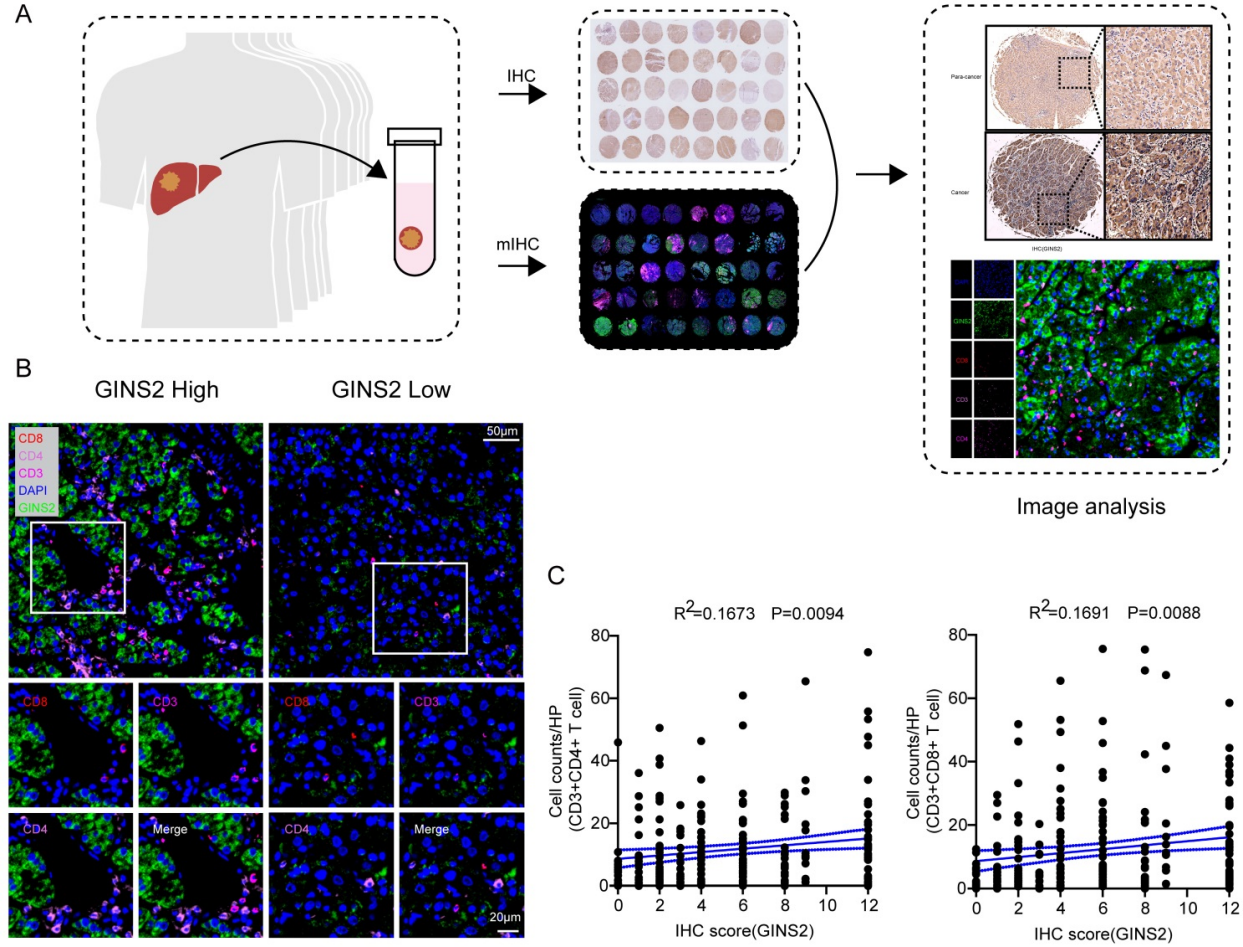
** Significant correlations between *GINS2* levels and CD3^+^CD8^+^ T cells/ CD3^+^CD4^+^ T cells in the validation cohort.** A. Schematic workflow of samples from validation cohort for traditional IHC and mIHC. B. Representative multiplex fluorescent immunohistochemistry images show the features of CD3^+^CD8^+^ T cells/CD3^+^CD4^+^ T cells within the tissues of high and low GINS2 expression. C. Correlation analyses of *GINS2* protein levels (IHC Score) and infiltration of CD3^+^CD8^+^ T cells and CD3^+^CD4^+^ T cells in the validation cohort.

To further explore the potential association between *GINS2* gene and different infiltrating immunocytes, we then utilized the TIMER and GEPIA databases to achieve this end. We investigated the associations between transcriptional levels of *GINS2* and gene markers of various immune cells or status and the results were demonstrated in Table 2. After equilibrating the effect of tumor purity, transcriptional levels of *GINS2* were positively related with most marker gene sets of CD8^+^ T cells, T cells (general) and Th1 cells in HCC (Table 2). We also analyzed the samples of validation cohort by multiplex immunohistochemistry (mIHC, Figure 5A). The protein levels of *GINS2* were positively associated with the number of infiltrating CD3^+^CD8^+^ T cells and CD3^+^CD4^+^ T cells (Figure 5B-C). To further elucidate the correlation between *GINS2* gene and T cell status, we examined the associations between *GINS2* levels and markers of activated T cells, including activated CD8^+^ T cells and CD4^+^ T cells (Table 3). After equilibrating the effect of tumor purity, the results revealed that the *GINS2* level showed positive correlation with most markers of activated T cells in HCC.

Among T cells, CD8^+^ T cells are the most significant effective anti-tumor cells. After CD8^+^ T cells infiltrate the tumor tissue, they gradually transition into a state of dysfunctional exhaustion under the chronic and continuous stimulation of tumor-related antigens, named T cell exhaustion, which is a significant mechanism in the weakening of antitumor effects 26. Interestingly, we noticed significant associations between *GINS2* levels and marker sets of Treg cells and T cell exhaustion, such as FOXP3, CCR8, TGFβ, PD-1, CTLA4, LAG3, and TIM-3 (Table 2). FOXP3 acts a pivotal role in Treg cells, in which it impedes the attack of cytotoxic T cells on cancerous cells 27. TIM-3, a vital gene that mediates T cell exhaustion, correlates positively with *GINS2* levels, implying that *GINS2* gene acts as a crucial role in TIM-3 mediating T cell exhaustion. Other immunosuppressive molecules, e.g., PD-1 and CTLA4, also showed important associations with *GINS2* levels. The above evidences revealed that there is a link between *GINS2* gene and T cell exhaustion and that *GINS2* may play a pivotal role in immune escape in the TME of HCC.

In addition, the transcriptional levels of most gene markers of TAMs, M1 macrophages, M2 macrophages and natural killer cells had weak or no correlations with *GINS2* expression (Table 2). We further verified the associations between *GINS2* gene and marker sets of monocytes and TAMs via the GEPIA database. The correlations between *GINS2* and markers of monocytes and TAMs were similar with those identified via TIMER database (Table 4). The findings suggest that *GINS2* does not exert much influence on the regulation of macrophage polarization in HCC.

## Discussion

DNA replication, an important step in the cell cycle enabling one single tumor cell to split into two, is emerging as an important factor in tumorigenesis and growth 28. GINS complex subunit 2 (*GINS2*), a crucial element of the DNA replication complex GINS, takes part in multiple processes of DNA replication 11. To gain new insights into the underlying roles of *GINS2* in liver cancer and its underlying mechanism, we applied a bioinformatic analysis based on public database. Analysis of the transcriptome in samples from 5 GEO datasets and 421 HCC samples from the TCGA database verified that transcriptional levels of *GINS2* are significantly elevated in cancerous tissues than in normal tissues, which is line with the results of the IHC staining of 236-patient validation cohort (Figure 1). Furthermore, *GINS2* gene reflected the prognosis of HCC patients. The overall survival, disease-free survival, progression-free survival, and relapse-free survival of patients with high-GINS2 expression were worse than those with low-GINS2 expression in the TCGA cohort.

High expression of *GINS2* can cause an unfavorable prognosis in male HCC patients. However, *GINS2* has different effects on the outcome of HCC cohorts of different ethnicities. High levels of *GINS2* can cause a poor prognosis in Asian HCC patients but cannot affect Caucasians. Additionally, our studies demonstrated that high *GINS2* levels displayed poor OS and DSS in patients without a history of alcohol consumption or hepatitis virus infection. Thus, our results hinted that *GINS2* might serve as a prognostic biomarker in HCC. In an attempt to determine underlying biological processes potentially responsible for the poor prognosis tied with *GINS2* expression, we found that *GINS2* gene was related with a series process of cell proliferation, say, cell cycle, DNA replication and cell division processes, in HCC. These processes affect tumor growth and the prognosis of hepatocellular carcinoma. Indeed, *GINS2*, as a novel unraveled oncogene, has been reported to affect cell viability, apoptosis, and cell cycle progression in pancreatic cancer 29. Knockdown of *GINS2* impairs cell proliferation and enhances apoptosis in non-small-cell lung cancer (NSCLC) 30. Given these direct and indirect effects of *GINS2*, we cautiously conclude that *GINS2* overexpression contributes to unfavorable prognosis in HCC patients and the underlying function of *GINS2* in HCC was correlated with DNA replication and cell cycle.

Our study also found that *GINS2* levels was correlated with the levels of diverse infiltrating immunocytes in HCC, which, until now, had not been extensively studied. Our analyses revealed that in HCC, the abundance and gene markers of diverse immunocytes were associated with the *GINS2* levels. Specifically, *GINS2* expression was related with the levels of infiltrating B cells, CD8^+^ T cells, macrophages, neutrophils, and dendritic cells (DCs) in HCC. Meanwhile, *GINS2* levels was significantly associated with marker sets of CD8^+^ T cells, T cells (general), Th1 cells, Treg cells and T cell exhaustion in HCC. Importantly, our results demonstrated that the levels of *GINS2* expression was positively correlated with most gene markers of activated T cells, especially CD8^+^ T cells, in HCC. CD8^+^ T cells, as the prime anti-tumor cells, can destroy cancerous cells by secreting perforin and granzyme B through the Fas/FasL pathway once they contact tumor cells or release IFN-γ and TNFα to eliminate tumor cells 2. However, CD8^+^ T lymphocytes in TME are often exhausted, which is tightly related to the activation of immune checkpoints (such as PD1 and CTLA4), as has been reported in our studies. Most malignant tumors, including HCC, enhance the expression of inhibitory ligands to get out of the immune response by destroying T cell function, thus lead to tumor progression. This is the crucial mechanism promoting tumor progression and immune escape. Moreover, the markers of Treg cells, FOXP3, CCR8 and TGF-β, are positively correlated with *GINS2* expression. Treg cells are one of the important subgroups of CD4^+^ T cells. Previous research has reported that as the tumor progresses, the number of Treg cells increases 31. Treg cells are important immunosuppressive cells, suggesting that HCC patients with high *GINS2* expression might have certain degrees of immunosuppression. TAMs can assist cancerous cells in various ways, e.g., promoting tumor cell proliferation, angiogenesis, immune escape, and metastasis 32-35. However, our results demonstrate that there are no or only weak associations between the levels of *GINS2* and marker sets of monocytes, TAMs, M1 macrophages, M2 macrophages and natural killer cells in HCC.

In conclusion, our study suggests that *GINS2* is a potential prognostic biomarker for HCC patients and is associated with the abundance of infiltrating immune cells in tumor tissues. Relatively high levels of *GINS2* in HCC may indicate a greater risk of poor prognosis, and considering the differences in infiltration levels of immune cells between the groups with high- and low- expression of *GINS2*, HCC patients with high *GINS2* expression may benefit from more accurate immunotherapy strategies.

## Supplementary Material

Supplementary figures and tables.

## Figures and Tables

**Table 1:** Clinicopathological characteristics in relation to GINS2 expression status in TCGA cohort.

Characteristics	TCGA Cohort (N=371)	GINS2 expression			
		High N=185(%)	Low N=186(%)	χ2	*P* value
**Age**					
>=70 y		145(50.3)	143(49.7)	0.120	0.729
<70 y		40(48.2)	43(51.8)		
**Gender**					
male		123(49.2)	127(50.8)	0.136	0.713
female		62(51.2)	59(48.8)		
**Race**					
ASIAN		92(58.2)	66(41.8)	7.836	0.098
BLACK or AFRICAN AMERICAN		7(41.2)	10(58.8)		
WHITE		81(44.0)	103(56.0)		
AMERICAN INDIAN or ALASKA NATIVE		1(50)	1(50)		
NA		4(40)	6(60))		
**T**					
T1-T2		126(45.7)	140(54.3)	7.653	0.006*
T3-T4		59(62.1)	36(37.9)		
**N**					
N0		133(52.8)	119(47.2)	4.494	0.213
N1		1(25)	3(75)		
NX		50(43.9)	64(56.1)		
NA		1(100)	0(0)		
**M**					
M0		139(52.3)	127(47.7)	2.737	0.255
M1		1(25)	3(75)		
MX		45(44.6)	56(55.4)		
**Pathologic Stage**					
stage I-II		117(45.3)	141(54.7)	7.651	0.022*
stage III-IV		56(62.2)	34(37.8)		
NA		12(52.2)	11(47.8)		

Statistical significance was determined by Chi-square test or Fisher's exact test. * *P* < 0.05

**Table 2:** Correlation analysis between GINS2 and related gene markers of immune cells in TIMER.

Description	Gene markers	LIHC			
		None		Purity	
		Cor	P	Cor	P
CD8^+^ T cell	CD8A	0.131	1.17E-02*	0.268	4.37E-07*
	CD8B	0.167	1.26E-03*	0.296	2.01E-08*
T cell(general)	CD3D	0.221	1.82E-05*	0.371	1.05E-12*
	CD3E	0.082	1.14E-01	0.259	1.12E-06*
	CD2	0.114	2.79E-02*	0.279	1.42E-07*
B cell	CD19	0.162	1.79E-03*	0.255	1.66E-06*
	CD79A	0.060	2.48E-01	0.191	3.55E-04*
Monocyte	CD86	0.203	8.65E-05*	0.374	7.02E-13*
	CD115(CSF1R)	0.090	8.48E-02	0.249	2.71E-06*
TAM	CCL2	0.011	8.29E-01	0.145	6.80E-03*
	CD68	0.078	1.34E-01	0.180	7.79E-04*
	IL10	0.120	2.07E-02*	0.248	3.24E-06*
M1 Macrophage	INOS(NOS2)	-0.002	9.76E-01	0.007	8.93E-01
	IRF5	0.345	7.81E-12*	0.348	2.76E-11*
	COX2(PTGS2)	-0.020	6.96E-01	0.119	2.77E-02*
M2 Macrophage	CD163	0.025	6.35E-01	0.154	4.21E-03*
	VSIG4	0.065	2.09E-01	0.199	2.03E-04*
	MS4A4A	0.029	5.80E-01	0.176	1.01E-03*
Neutrophils	CD66b(CEACAM8)	0.078	1.35E-01	0.120	2.62E-02*
	CD11b(ITGAM)	0.272	1.13E-07*	0.378	3.89E-13*
	CCR7	-0.027	6.02E-01	0.127	1.85E-02*
Natural Killer cell	KIR2DL1	-0.028	5.95E-01	-0.044	4.13E-01
	KIR2DL3	0.116	2.53E-02*	0.157	3.53E-03*
	KIR2DL4	0.191	2.09E-04*	0.230	1.58E-05*
	KIR3DL1	-0.002	9.65E-01	0.008	8.79E-01
	KIR3DL2	0.049	3.43E-01	0.100	6.36E-02
	KIR3DL3	0.000	9.92E-01	0.014	8.00E-01
	KIR2DS4	0.036	4.94E-01	0.032	5.53E-01
Dendritic cell	HLA-DPB1	0.130	1.24E-02	0.274	2.38E-07*
	HLA-DQB1	0.145	5.19E-03*	0.288	5.03E-08*
	HLA-DRA	0.110	3.43E-02*	0.247	3.31E-06*
	HLA-DPA1	0.090	8.32E-02	0.236	9.70E-06*
	BDCA-1(CD1C)	-0.026	6.13E-01	0.083	1.22E-01
	BDCA-4(NRP1)	0.058	2.65E-01	0.088	1.04E-01
	CD11c(ITGAX)	0.194	1.75E-04*	0.335	1.71E-10*
Th1 cell	T-bet(TBX21)	0.013	8.03E-01	0.134	1.25E-02*
	STAT4	0.173	8.59E-04*	0.252	2.22E-06*
	STAT1	0.249	1.31E-06*	0.305	7.48E-09*
	IFN-y(IFNG)	0.221	1.77E-05*	0.310	3.88E-09*
	TNF-a(TNF)	0.149	4.12E-03*	0.289	4.88E-08*
Th2 cell	GATA3	0.102	4.92E-02*	0.253	1.91E-06*
	STAT6	0.038	4.60E-01	0.026	6.36E-01
	STAT5A	0.240	2.99E-06*	0.306	6.77E-09*
	IL13	0.123	1.75E-02*	0.123	2.27E-02*
Tfh cell	BCL6	0.011	8.31E-01	0.015	7.79E-01
	IL21	0.078	1.34E-01	0.130	1.58E-02*
Th17 cell	STAT3	0.030	5.61E-01	0.076	1.61E-01
	IL17A	0.001	9.84E-01	0.019	7.28E-01
Treg cell	FOXP3	0.116	2.52E-02*	0.202	1.53E-04*
	CCR8	0.203	8.53E-05*	0.297	1.82E-08*
	STAT5B	0.135	9.18E-03*	0.103	5.68E-02
	TGFβ(TGFB1)	0.138	7.79E-03*	0.241	6.03E-06*
T cell exhaustion	PD-1(PDCD1)	0.217	2.38E-05*	0.340	8.68E-11*
	CTLA4	0.230	7.77E-06*	0.362	3.90E-12*
	LAG3	0.292	1.16E-08*	0.356	9.35E-12*
	TIM-3(HAVCR2)	0.229	8.81E-06*	0.404	5.29E-15*

TIMER: Correlation module analyzes the expression between a pair of specific genes in HCC, together with the Spearman's rho value and estimated statistical significance. Options for partial correlation conditioned on tumor purity are also provided. Cor, R value of Spearman's correlation; None, correlation without adjustment. Purity, correlation adjusted by purity. * P < 0.05.

**Table 3:** Correlation analysis between GINS2 and markers of activated T cells in HCC patients in TIMER.

Activated CD8Tcell	None		Purity		Activated CD4Tcell	None		Purity	
	Cor	P	Cor	P		Cor	P	Cor	P
ADRM1	0.386	1.36e-14*	0.384	1.53E-13*	AIM2	0.187	2.87e-04*	0.331	2.89E-10*
AHSA1	0.28	4.77e-08*	0.309	4.41E-09*	BIRC3	0.162	1.73e-03*	0.230	1.58E-05*
C1GALT1C1	0.132	1.11e-02*	0.161	2.69E-03*	BRIP1	0.525	1.00e-27*	0.511	2.63E-24*
CCT6B	-0.121	1.93e-02*	-0.139	9.80E-03	CCL20	0.121	2.02e-02*	0.146	6.58E-03*
CD37	0.122	1.88e-02*	0.310	4.21E-09*	CCL4	0.112	3.17e-02*	0.264	6.71E-07*
CD3D	0.221	1.82e-05*	0.371	1.05E-12*	CCL5	0.129	1.27e-02*	0.278	1.54E-07*
CD3E	0.082	1.14e-01	0.259	1.12E-06*	CCNB1	0.75	2.72e-68*	0.742	1.91E-61*
CD3G	0.102	4.97e-02*	0.214	6.39E-05*	CCR7	-0.027	6.02e-01	0.127	1.85E-02*
CD69	0.02	6.97e-01	0.159	3.07E-03*	DUSP2	0.178	5.88e-04*	0.299	1.42E-08*
CD8A	0.131	1.17e-02*	0.268	4.37E-07*	ESCO2	0.689	0.00e+00*	0.678	1.04E-47*
CETN3	0.35	3.83e-12*	0.326	5.68E-10*	ETS1	-0.034	5.15e-01	0.071	1.88E-01
CSE1L	0.526	0.00e+00*	0.515	9.85E-25*	EXO1	0.701	0.00E+00*	0.742	1.32E-75*
GEMIN6	0.362	6.27e-13*	0.334	1.92E-10*	EXOC6	0.327	1.46e-10*	0.313	2.80E-09*
GNLY	0.093	7.37e-02	0.168	1.71E-03*	IARS	0.352	3.78e-12*	0.352	1.69E-11*
GPT2	-0.229	8.73e-06*	-0.231	1.41E-05*	ITK	0.033	5.29e-01	0.186	5.22E-04*
GZMA	0.009	7.59e-02	0.223	2.90E-05*	KIF11	0.762	1.57e-71*	0.749	3.52E-63*
GZMH	0.051	3.26e-01	0.134	1.30E-02*	KNTC1	0.754	0.00e+00*	0.736	4.62E-60*
GZMK	0.014	7.91e-01	0.165	2.15E-03*	NUF2	0.753	0.00e+00*	0.741	3.38E-61*
IL2RB	0.15	3.72e-03*	0.290	4.16E-08*	PRC1	0.755	0.00e+00*	0.739	6.77E-61*
LCK	0.103	4.73e-02*	0.268	4.30E-07*	PSAT1	0.143	5.79e-03*	0.148	6.05E-03*
MPZL1	0.386	1.21e-14*	0.414	1.04E-15*	RGS1	0.223	1.50e-05*	0.363	3.62E-12*
NKG7	0.007	1.64e-01	0.173	1.26E-03*	RTKN2	0.603	3.67e-38*	0.588	2.05E-33*
PIK3IP1	-0.01	8.42e-01	0.031	5.65E-01	SAMSN1	0.177	6.48e-04*	0.356	9.15E-12*
PTRH2	0.228	1.01e-05*	0.230	1.58E-05*	SELL	0.072	1.67e-01	0.189	4.05E-04*
TIMM13	0.252	8.91e-07*	0.243	4.87E-06*	TRAT1	0.011	8.32e-01	0.153	4.29E-03*
ZAP70	0.07	1.79e-01	0.207	1.05E-04*					

TIMER: Correlation module analyzes the expression between a pair of specific genes in LIHC, together with the Spearman's rho value and estimated statistical significance. Options for partial correlation conditioned on tumor purity are also provided. Cor, R value of Spearman's correlation; None, correlation without adjustment. Purity, correlation adjusted by purity. * *P* < 0.05.

**Table 4:** Correlation analysis between GINS2 and related gene markers of monocyte and macrophages in GEPIA.

Description	Gene markers	HCC			
		Tumor		Normal	
		R	*P*	R	*P*
Monocyte	CD86	0.18	6.20E-04*	0.056	7.00E-01
	CD115(CSF1R)	0.12	2.30E-02*	0.059	6.80E-01
TAM	CCL2	0.028	5.90E-01	0.076	6.00E-01
	CD68	0.05	3.40E-01	0.012	9.40E-01
	IL10	0.16	2.50E-03*	0.23	1.10E-01
M1 Macrophage	INOS (NOS2)	-0.0082	8.80E-01	0.58	1.20E-05*
	IRF5	0.26	3.50E-07*	0.095	5.10E-01
	COX2(PTGS2)	-0.0081	8.80E-01	-0.024	8.70E-01
M2 Macrophage	CD163	0.12	2.60E-02*	-0.09	5.30E-01
	VSIG4	0.11	3.20E-02*	-0.085	5.60E-01
	MS4A4A	0.083	1.10E-01	0.021	8.80E-01

R. Value of Pearson's correlation; *P*. Statistical significance. * *P* < 0.05.
