# Supplementary material for: Identification of GINS2 prognostic potential and involvement in immune cell infiltration in hepatocellular carcinoma
Source: J Cancer. 2022 Jan 1;13(2):610–22. doi: 10.7150/jca.53841 (PMC8771526; doi:10.7150/jca.53841)
Supplement: Supplementary file 1 — Supplementary figures and tables. [file jcav13p0610s1.pdf]

Figure S1. Up-regulated *GINS2* in tumorous tissues and predicts poorer prognoses of HCC patients. A. Transcriptional levels of *GINS2* in diverse solid tumor from TCGA database.

Figure S2. Subgroup survival plots of *GINS2*-high and *GINS2*-low expression HCC patients in the TCGA dataset. A. Overall survival (OS) analyses subgrouped by gender, race, alcohol consumption and hepatitis infection. B. Disease-specific survival (DSS) curves plotted by gender, race, alcohol consumption and hepatitis infection.

Figure S3. KEGG analysis of down-regulated differential expressed genes grouped by *GINS2* expression. Pathway enrichment (A) and biological process (B) of 280 down-regulated genes by comparing *GINS2*-high and *GINS2*-low samples from the TCGA database.

Figure S4. Associations between *GINS2* and DNA replication related genes in HCC. A. GSEA analysis hints *GINS2* is positively associated with DNA replication. B. Correlation analyses of *GINS2* expression and levels of DNA replication related genes.

A

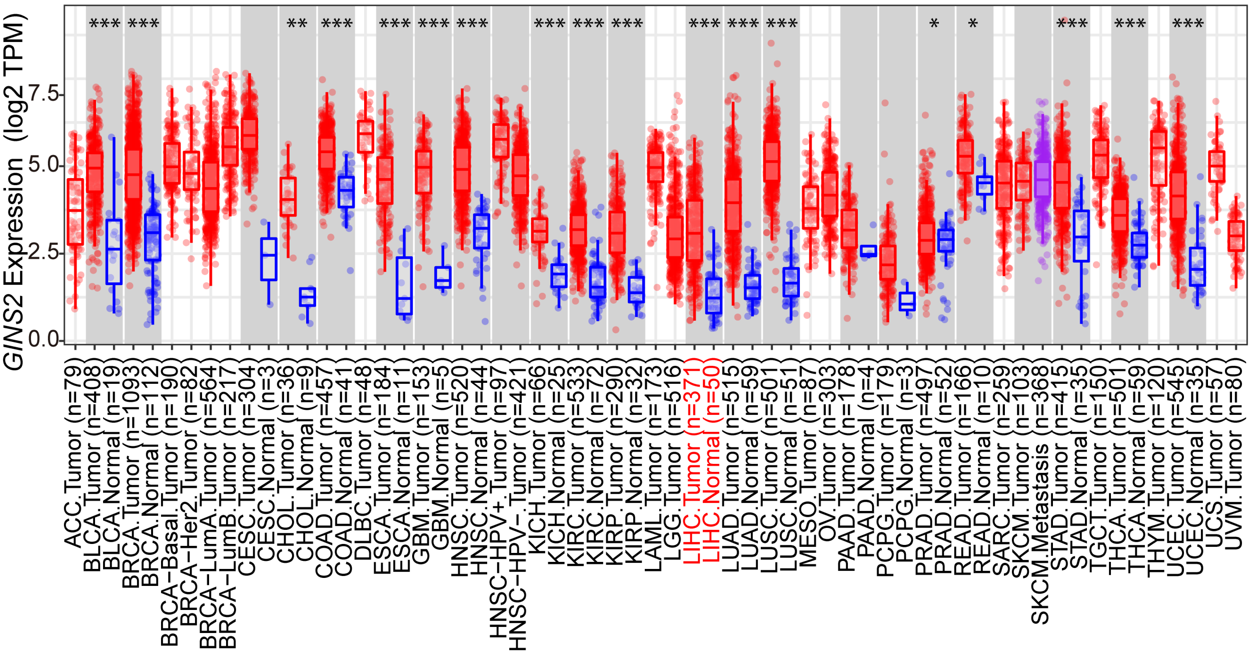
*** *** ** *** *** *** *** *** *** *** *** *** *** * * *** *** ***
GINS2 Expression (log2 TPM)
0.0
2.5
5.0
7.5
ACC.Tumor (n=79)
BLCA.Tumor (n=408)
BLCA.Normal (n=19)
BRCA.Tumor (n=1093)
BRCA.Normal (n=112)
BRCA−Basal.Tumor (n=190)
BRCA−Her2.Tumor (n=82)
BRCA−LumA.Tumor (n=564)
BRCA−LumB.Tumor (n=217)
CESC.Tumor (n=304)
CESC.Normal (n=3)
CHOL.Tumor (n=36)
CHOL.Normal (n=9)
COAD.Tumor (n=457)
COAD.Normal (n=41)
DLBC.Tumor (n=48)
ESCA.Tumor (n=184)
ESCA.Normal (n=11)
GBM.Tumor (n=153)
GBM.Normal (n=5)
HNSC.Tumor (n=520)
HNSC.Normal (n=44)
HNSC−HPV+.Tumor (n=97)
HNSC−HPV−.Tumor (n=421)
KICH.Tumor (n=66)
KICH.Normal (n=25)
KIRC.Tumor (n=533)
KIRC.Normal (n=72)
KIRP.Tumor (n=290)
KIRP.Normal (n=32)
LAML.Tumor (n=173)
LGG.Tumor (n=516)
LIHC.Tumor (n=371)
LIHC.Normal (n=50)
LUAD.Tumor (n=515)
LUAD.Normal (n=59)
LUSC.Tumor (n=501)
LUSC.Normal (n=51)
MESO.Tumor (n=87)
OV.Tumor (n=303)
PAAD.Tumor (n=178)
PAAD.Normal (n=4)
PCPG.Tumor (n=179)
PCPG.Normal (n=3)
PRAD.Tumor (n=497)
PRAD.Normal (n=52)
READ.Tumor (n=166)
READ.Normal (n=10)
SARC.Tumor (n=259)
SKCM.Tumor (n=103)
SKCM.Metastasis (n=368)
STAD.Tumor (n=415)
STAD.Normal (n=35)
TGCT.Tumor (n=150)
THCA.Tumor (n=501)
THCA.Normal (n=59)
THYM.Tumor (n=120)
UCEC.Tumor (n=545)
UCEC.Normal (n=35)
UCS.Tumor (n=57)
UVM.Tumor (n=80)

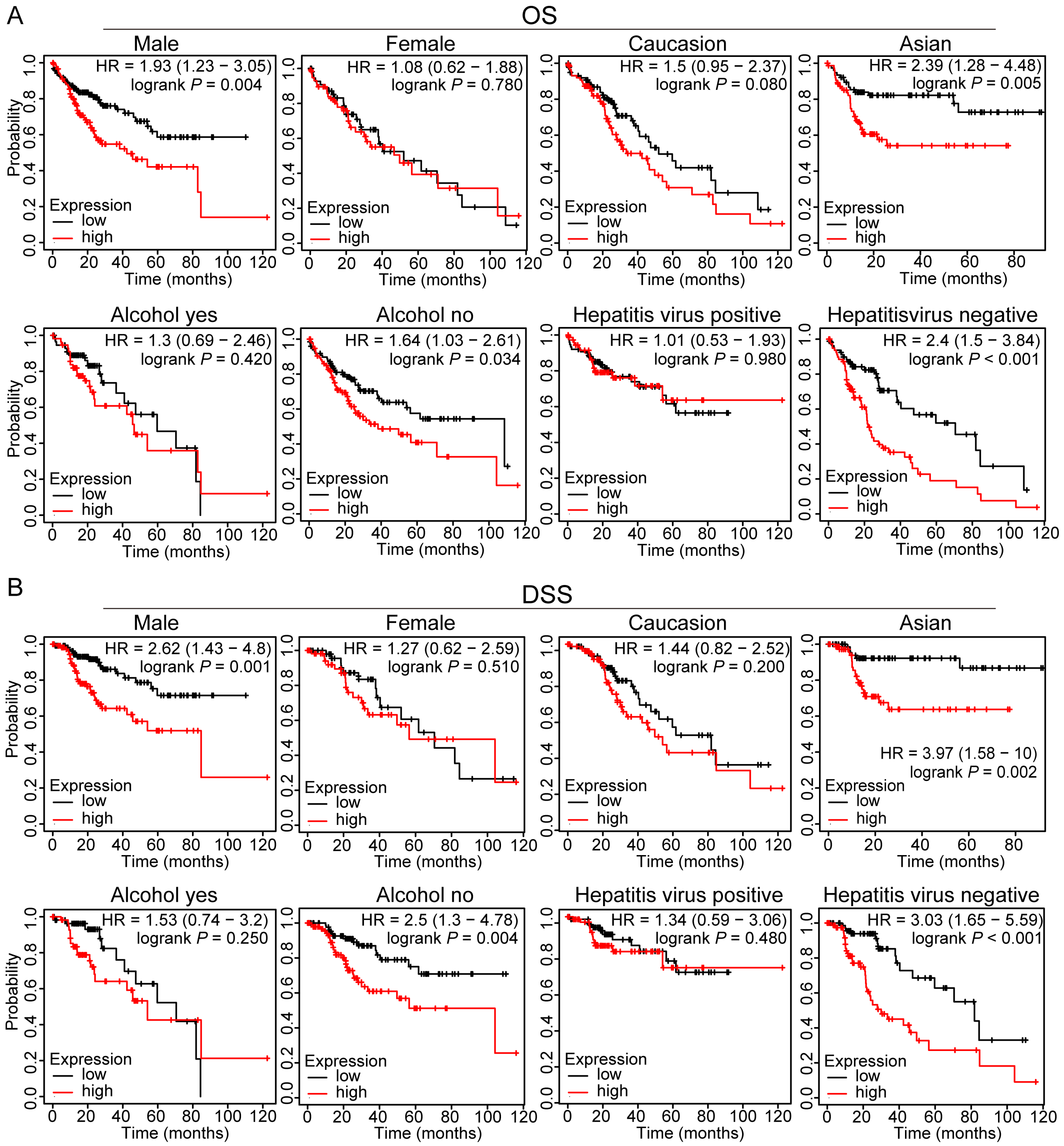
A
OS
Male
HR = 1.93 (1.23 − 3.05)
logrank P = 0.004
Female
HR = 1.08 (0.62 − 1.88)
logrank P = 0.780
Caucasion
HR = 1.5 (0.95 − 2.37)
logrank P = 0.080
Asian
HR = 2.39 (1.28 − 4.48)
logrank P = 0.005
Alcohol yes
HR = 1.3 (0.69 − 2.46)
logrank P = 0.420
Alcohol no
HR = 1.64 (1.03 − 2.61)
logrank P = 0.034
Hepatitis virus positive
HR = 1.01 (0.53 − 1.93)
logrank P = 0.980
Hepatitisvirus negative
HR = 2.4 (1.5 − 3.84)
logrank P < 0.001
B
DSS
Male
HR = 2.62 (1.43 − 4.8)
logrank P = 0.001
Female
HR = 1.27 (0.62 − 2.59)
logrank P = 0.510
Caucasion
HR = 1.44 (0.82 − 2.52)
logrank P = 0.200
Asian
HR = 3.97 (1.58 − 10)
logrank P = 0.002
Alcohol yes
HR = 1.53 (0.74 − 3.2)
logrank P = 0.250
Alcohol no
HR = 2.5 (1.3 − 4.78)
logrank P = 0.004
Hepatitis virus positive
HR = 1.34 (0.59 − 3.06)
logrank P = 0.480
Hepatitis virus negative
HR = 3.03 (1.65 − 5.59)
logrank P < 0.001
Expression
low
high
Probability
Time (months)

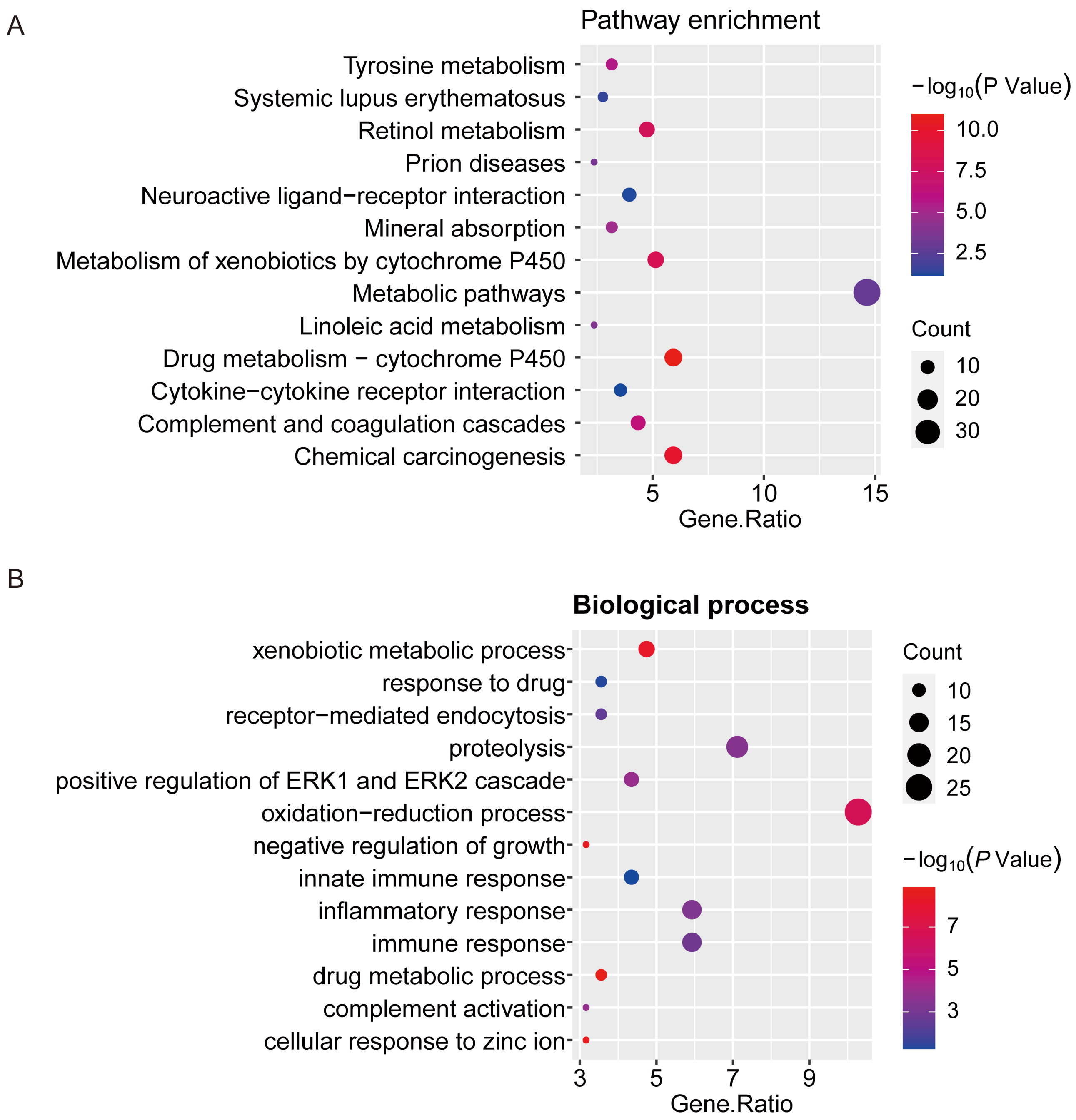
A
Pathway enrichment
Tyrosine metabolism
Systemic lupus erythematosus
Retinol metabolism
Prion diseases
Neuroactive ligand−receptor interaction
Mineral absorption
Metabolism of xenobiotics by cytochrome P450
Metabolic pathways
Linoleic acid metabolism
Drug metabolism − cytochrome P450
Cytokine−cytokine receptor interaction
Complement and coagulation cascades
Chemical carcinogenesis
5
10
15
Gene.Ratio
−log10(P Value)
10.0
7.5
5.0
2.5
Count
10
20
30
B
Biological process
xenobiotic metabolic process
response to drug
receptor−mediated endocytosis
proteolysis
positive regulation of ERK1 and ERK2 cascade
oxidation−reduction process
negative regulation of growth
innate immune response
inflammatory response
immune response
drug metabolic process
complement activation
cellular response to zinc ion
3
5
7
9
Gene.Ratio
Count
10
15
20
25
−log10(P Value)
7
5
3

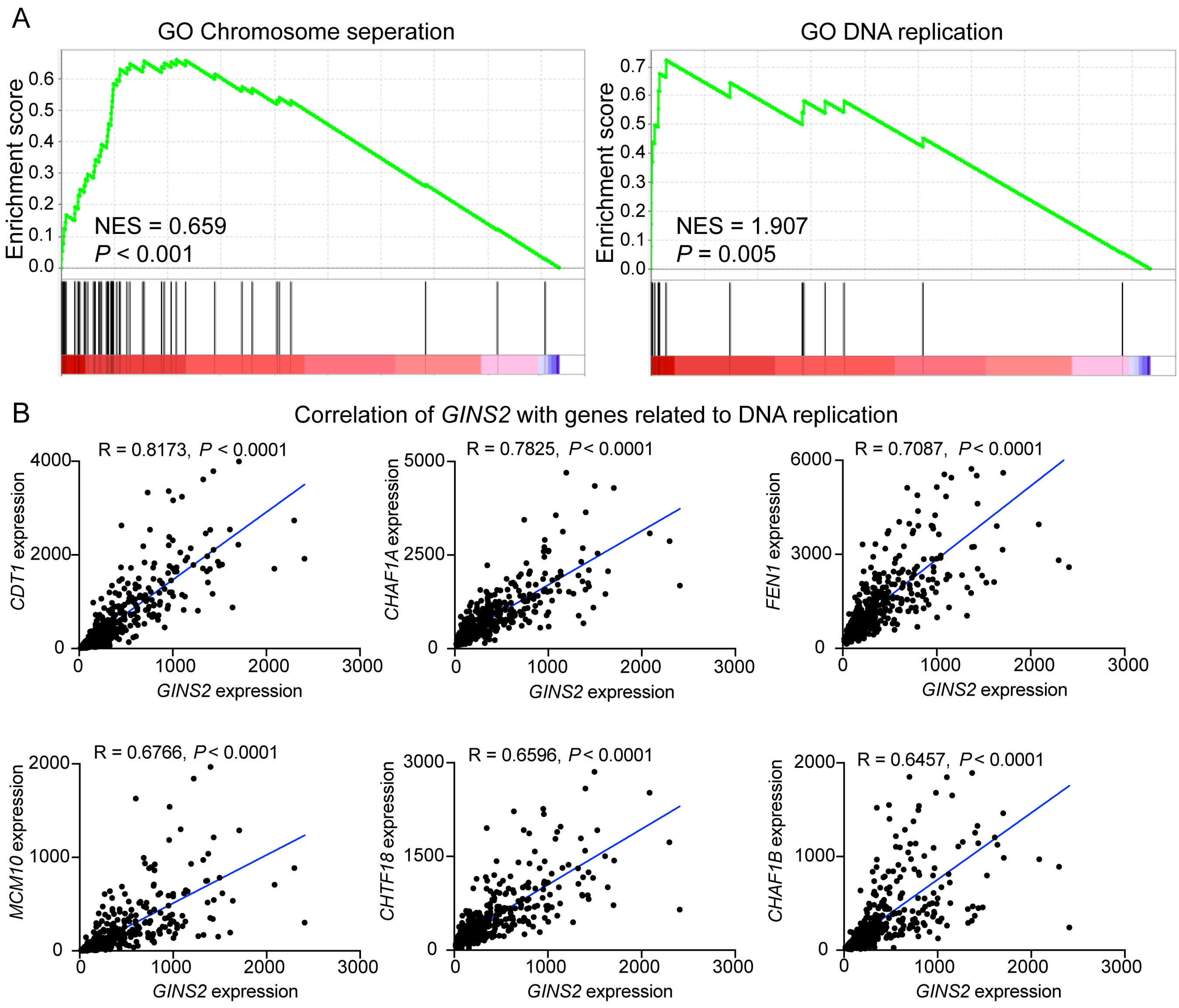
A
GO Chromosome seperation
Enrichment score
NES = 0.659
P < 0.001
GO DNA replication
NES = 1.907
P = 0.005
B
Correlation of GINS2 with genes related to DNA replication
R = 0.8173, P < 0.0001
CDT1 expression
GINS2 expression
R = 0.7825, P< 0.0001
CHAF1A expression
R = 0.7087, P< 0.0001
FEN1 expression
R = 0.6766, P< 0.0001
MCM10 expression
R = 0.6596, P< 0.0001
CHTF18 expression
R = 0.6457, P< 0.0001
CHAF1B expression

Table S1. Details of GEO series included in this study.

GEO series	Contributors	Nontumor	Tumor	Platform
GSE77314	Liu G, Hou G, Li L, Li Y et al.2016	50	50	Illumina Genome Analyzer (Homo sapiens)
GSE45436	Wang HW, Hsieh TH, Huang SY, et al.2013	39	95	Affymetrix Human Genome U133 Plus 2.0 Array
GSE36376	Lim HY, Sohn I, Deng S, Lee J et al.2013	193	249	Illumina HumanHT-12 V4.0 expression beadchip
GSE25097	Zhang C.et al.2011	243	268	Rosetta/Merck Human RSTA Affymetrix 1.0 microarray, Custom CD
GSE14520	Roessler S, Jia HL, Budhu A, Forgues M et al.2010	220	225	Affymetrix HT Human Genome U133A Array

Table S2. Clinicopathological characteristics in relation to GINS2 expression status in validation cohort.

characteristics	Case (N=236)	IHC score		χ2	*P* value
		<6	>=6		
**Age**					
>=70 y		10(90.9)	1(9.1)	6.692	0.010*
<70y		104(46.2)	121(53.8)		
**Gender**					
male		99(48.1)	107(51.9)	0.040	0.842
female		15(50)	15(50)		
**History of hepatitis**					
Yes		86(50.9)	83(49.1)	1.590	0.207
no		28(41.8)	39(58.2)		
**Status**					
Live		84(53.2)	74(46.8)	4.521	0.033
dead		30(38.5)	48(61.5)		
**Tumor Thrombus**					
Yes		38(42.2)	52(57.8)	2.156	0.142
none		76(52.1)	70(47.9)		
**Pathologic Stage**					
**I-II**		79(48.5)	84(51.5)	0.005	0.941
**III-IV**		35(47.9)	38(52.1)		
**T**					
T1-T2		98(50.5)	96(49.5)	2.133	0.144
T3-T4		16(38.1)	26(61.9)		
**N**					
N0		114(49.4)	117(50.6)	3.002	0.061
N1		0(0)	5(100)		
**M**					
M0		113(48.3)	121(51.7)	<0.01	1.000
M1		1(50)	1(50)		
**TMN Stage**					
**I-II**		97(51.6)	91(48.4)	4.008	0.045*
**III-IV**		17(35.4)	31(64.6)		

Statistical significance was determined by Chi-square *test or* Fisher*'s exact test. * P < 0.05*
